# Olive leaf spot caused by *Venturia oleaginea*: An updated review

**DOI:** 10.3389/fpls.2022.1061136

**Published:** 2023-01-09

**Authors:** Roberto Buonaurio, Leen Almadi, Franco Famiani, Chiaraluce Moretti, Giovanni Enrico Agosteo, Leonardo Schena

**Affiliations:** ^1^ Dipartimento di Scienze Agrarie, Alimentari e Ambientali, Università degli Studi di Perugia, Perugia, Italy; ^2^ Dipartimento di Agraria, Università Mediterranea di Reggio Calabria, Reggio Calabria, Italy

**Keywords:** control, Fusicladium oleagineum, Olea europaea, peacock’s eye disease, *Spilocaea oleagina*

## Abstract

Olive leaf spot (OLS) caused by *Venturia oleaginea* is widespread in all olive-growing areas and continents, where can cause severe yield losses. The disease is often underestimated for the difficulty to reveal early leaf symptoms and for the pathogen-induced phylloptosis, which creates the illusion of healthy and restored plants. The present review provide updated information on taxonomy, pathogen life style and cycle, epidemiology, diagnosis, and control. Application of copper-based fungicides is the main method to control OLS. However, the regulation 2009/1107 of the European Commission include these fungicides in the list of substances candidates for substitution. It is therefore urgent to find alternative control strategies especially for organic agriculture. Among new approaches/strategies for controlling OLS, promising results have been obtained using nanotechnology, endophytic microbes, and biostimulants.

## 1 Introduction

Olive leaf spot (OLS) caused by the ascomycetous fungus *Venturia oleaginea* (Castagne) Rossman & Crous (the anamorph: *Spilocaea oleagina*), also known as peacock’s eye disease, was described for the first time in France (Marseille) by Castagne (1845). The disease is widespread in the Mediterranean regions and in all olive (*Olea europaea*)-growing areas and continents, where can cause severe yield losses (Agosteo and Schena, 2011).

**Figure 1:**
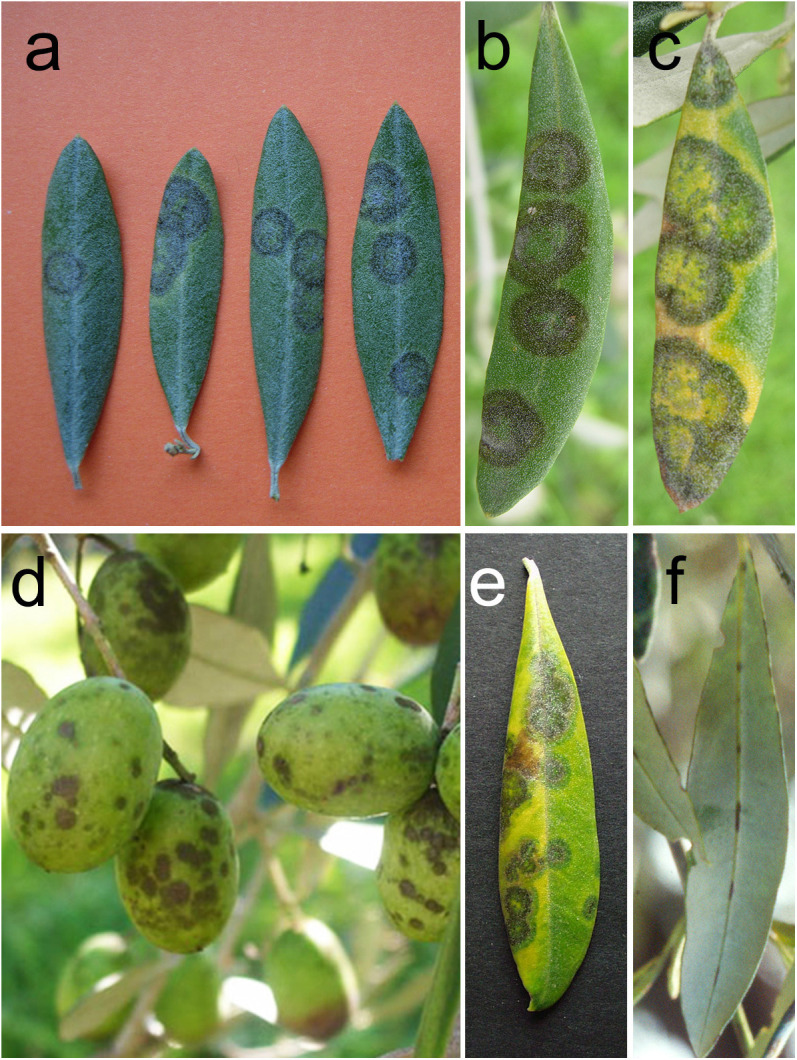
Symptoms and signs caused by *Venturia oleaginea* in olive trees. **(A)** circular spots uniformly coloured in brown green on upper leaf surface; **(B)** concentric rings which are olive-green, grey, dark brown from center to periphery on upper leaf surface; **(C)** yellow haloes surrounding the leaf spots; **(D)** brown velvety spots on olive drupes; **(E)** green islands surrounding the leaf spots; **(F)** dark brown streaks on the main vein of the lower leaf surface.

Typical diseases symptoms (**Figure 1**) appear on the upper side of the leaves and much less frequently on the lower side (Issa et al., 2019). Symptoms on shoots, leaf stalks, fruit pedicels and peduncles, fruits and inflorescences are rare. The first symptoms on upper leaf surface are characterized by circular brown green spots (2-10 mm in diameter), which are barely visible as their colour is similar to that of the healthy surrounding areas. Successively, the spots usually developing in concentric rings, which are olive-green, grey, dark brown from center to periphery. With the eruption of conidiophores and conidia, the spots assume a velvety appearance. In the most severe cases, spots can coalesce. On the lower leaf surface, dark brown streaks are evident on the main vein while the abundant presence of peltate trichomes in the rest of the lamina masks symptoms (**Figure 1F**). During summer, the leaf portions not interested by the spots become chlorotic and yellow; sometimes dark green halos where senescence is retarded (green islands) surround the spots (**Figure 1E**). Severe and recurrent attacks of the fungus provoke an intense defoliation and poor growth and dieback of defoliated branches. The incidence of the disease and the consequent defoliation is more severe in the lower part of the tree canopy (Issa et al., 2019).

Sometimes small brown sunken spots appear on tender shoots and fruit pedicels leading to early fruit drop or shrivelling. During very rainy periods in late summer and fall, small brown spots, sometimes surrounded by a reddish halo, can appear on drupe especially when they are green or, more frequently, near the maturity (**Figure 1D**). Attacked drupes wrinkle and become deformed due to the stumped growth of the infected tissues.

The defoliation caused by the fungus has a direct negative impact on the length of inflorescences, on fruit set and, consequently, on plant growth and productivity (Agosteo and Zappia, 2007; Issa et al., 2019). In general, an average yield loss of 20-30% has been estimated in areas where OLS is recurrent (Agosteo and Schena, 2011).

## 2 The causal agent

The causal agent of OLS disease was first named *Cycloconium oleaginum* (Castagne, 1845) successively placed in the genus *Spilocaea* as *Spilocaea oleagina* (Hughes, 1953; Graniti, 1993) and then in the genus *Fusicladium* as *Fusicladium oleagineum* (Schubert et al., 2003). Recently, the name *Venturia oleaginea* (Castagne) Rossman & Crous has been proposed according to the phylogenetic collocation of the fungus and the auspices of the International Commission on the Taxonomy of Fungi (ICTF), which recommend one name for use among pleomorphic genera (Rossman et al., 2015). *V. oleaginea* belongs to the *Dothideomycetes* class, *Pleosporomycetidae* subclass, and *Venturiales* order and is considered a biotrophic fungus (Benitez et al., 2005). However, similarly to other phytopathogenic *Venturia* spp. (Gonzalez-Dominguez et al., 2017), it can be considered an hemibiotrophic fungus as differently from true biotrophic fungi, which are not cultured *in vitro*, *V. oleaginea* is, albeit with difficulty (Petri, 1913; Saad and Masri, 1978; Viruega et al., 2011). This hypothesis is strengthened by results of a recent meta-analysis which highlighted a significant relationship between the type of trophic interactions of fungi with their hosts and the duration of latent period, expressed in degree-days (DD) (Précigout et al., 2020). Necrotrophs exhibited the shortest latent periods <100 DD, biotrophs had intermediate ones (between 100 and 200 DD), and hemibiotrophs had the longest latent periods (>200 DD) (Précigout et al., 2020). *V. oleaginea* belong to this last group since it has a latent period ranging from 256 to more than 3000 DD (Gonzalez-Dominguez et al., 2017).

Sexual stage of *V. oleaginea* is not currently known (Graniti, 1993; Agosteo and Schena, 2011). It is worth noting that sexual stage (pseudothecia) of others phytopathogenic *Venturiales* is found in those attacking deciduous fruit trees such as apple, cherry, nectarine, European and Asian pears, or pecan but not in those species affecting evergreen trees, including pyracantha (*Fusicladium pirachantae*), loquat (*Fusicladium eriobotryae*), and olive (Viruega et al., 2013; Gonzalez-Dominguez et al., 2017; Charlton et al., 2020).

However, a recent study conducted in Uruguay suggested the presence of a mixed mode of reproduction since two genetically different populations were identified and both showed moderate gene diversity and linkage disequilibrium (Bernaschina et al., 2020). Furthermore, pseudoparenchymal spherical tissues similar to immature *Venturia* pseudothecia were observed in olive leaves infected by *V. oleaginea* (Viruega et al., 2013).

**Figure 2:**
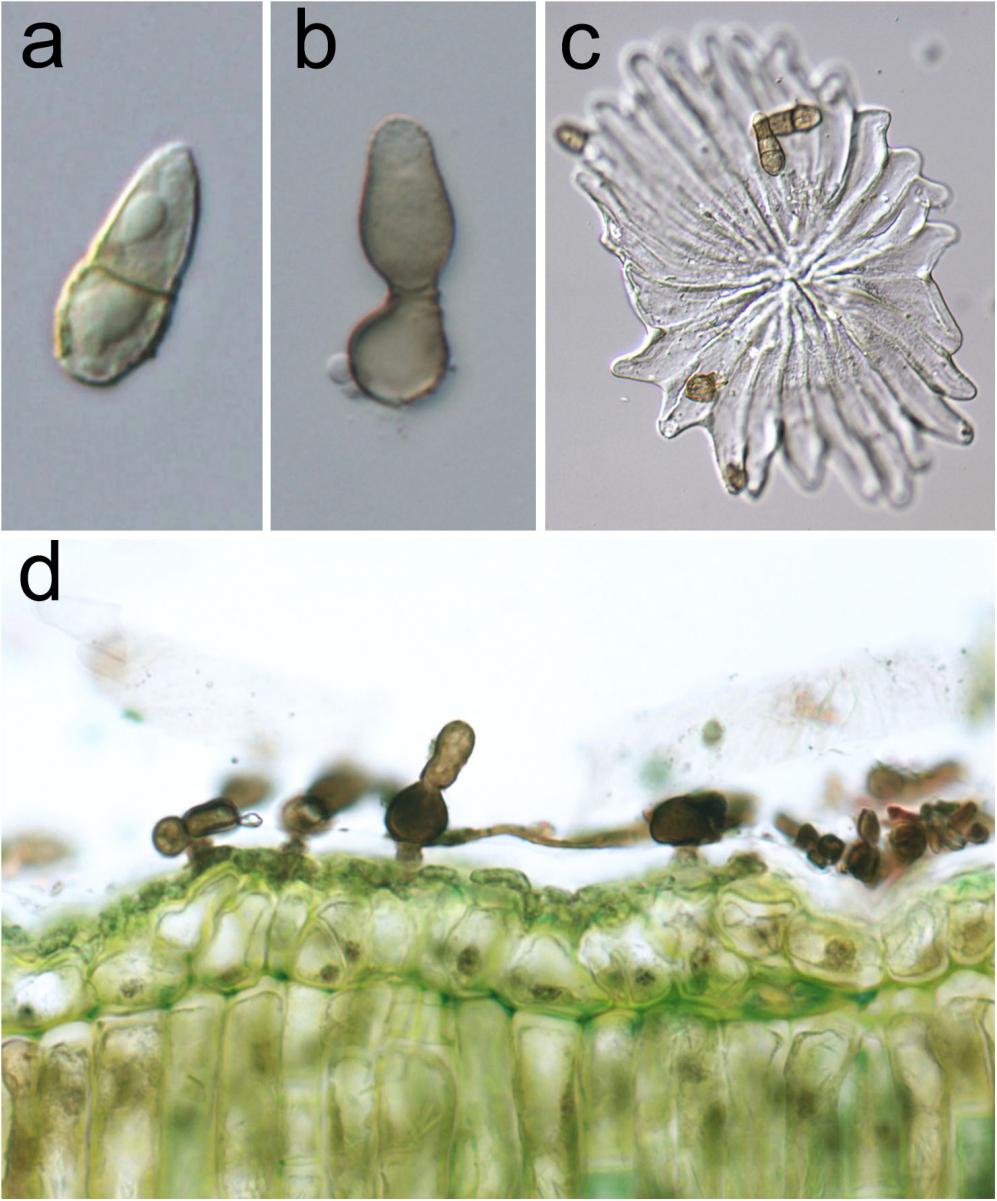
*Venturia oleaginea* anamorphic structures. **(A)** One-septate oval-pyriform conidium; **(B)** subglobose conidiophore producing a conidium at its apex, annellations are visible at the top of conidiogenous cell; **(C)** conidia on an olive peltate trichome (trichome represents an entry *via* as well as can function as parachute to spread the disease); **(D)** olive leaf section showing the presence of the mycelium in the cuticular layer.

The anamorph of *V. oleaginea* (**Figure 2**) is characterized by brown-olivaceous, oval-pyriform conidia truncate at the base and elongated at the top, 1-septate, sometimes 2-septate, often slightly constricted at the septum, measuring 15-30 x 9-15 μm (Schubert et al., 2003). Conidiophores are solitary, arising from hyphal cells, erumpent through the cuticle, subglobose, 8–10 µm in diameter, or ampulliform, 10–25 × 5–7 µm, or up to 15 µm wide at the base. They are erect, straight, unbranched, mostly aseptate, medium to dark brown, paler towards the apex, sometimes smooth, usually roughwalled, thick-walled. Conidiophores are reduced to conidiogenous cells, with a single or rarely with two or three conidiogenous loci, proliferation percurrent, with up to seven conspicuous annellations (Schubert et al., 2003).

The genome of a Chinese *V. oleaginea* strain has been recently sequenced by Jaber et al. (2020). It has a size of 46.08 Mb, a G/C content of 48.62%, approximately 23.02% of repetitive sequences and a total of 11,540 genes. Based on telomere representation, it was estimated that *V. oleaginea* has at least 16 chromosomes.

## 3 Life cycle and epidemiology

**Figure 3:**
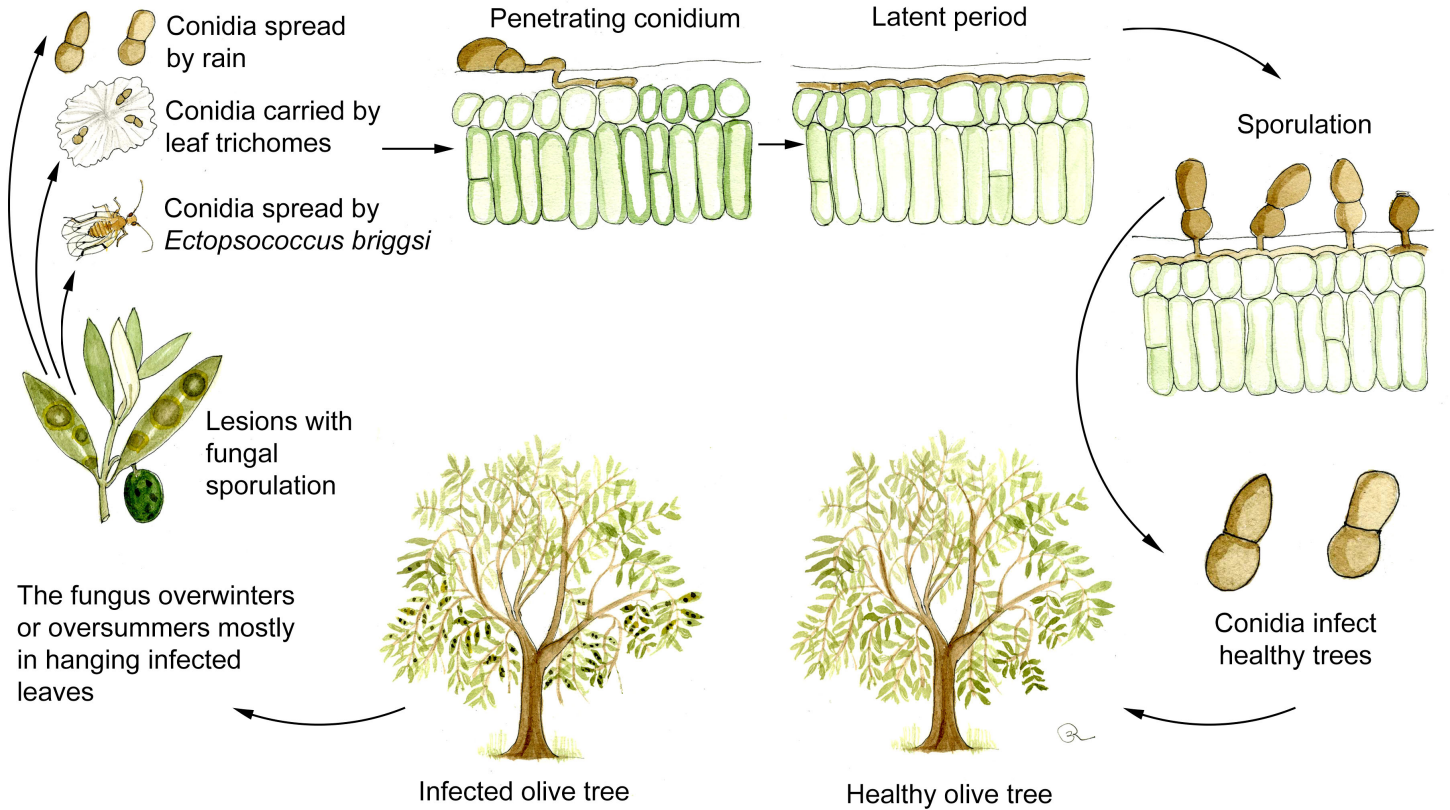
Disease cycle of olive leaf spot caused by *Venturia oleaginea* in olive trees. Figure drawn by Roberto Buonaurio.

Although as stated above, the existence of a sexual stage of *V. oleaginea* cannot be completely excluded (Viruega et al., 2013; Bernaschina et al., 2020) asexual conidia indeed constitute the most important source of inoculum. New infections are mainly caused by conidia produced on infected leaves still attached to the plant since detached leaves are quickly decomposed on the ground by saprotrophic microorganism (Agosteo and Schena, 2011; Viruega et al., 2013). Consequently, overwintering and oversummering of the pathogen mostly occur in hanging leaves as manifest and, more importantly, as latent infections (**Figure 3**).

*V. oleaginea* conidia are produced from 5 to 25°C and optimally under continuous wetness. At 70% RH sporulation is lower than 50% respect to those observed in optimal wetness conditions (Lawrence and Zehr, 1982; Obanor, 2006; Romero et al., 2018). Conidia are mainly dispersed by rain, while wind dissemination is poorly effective reaching maximum 40 m from infected trees (Lops et al., 1993; Frisullo et al., 1994; Viruega and Trapero, 1999). Wind dissemination can be facilitated when conidia are attached to leaf trichomes (**Figure 2C**), which work like a parachute when detached from the leaves (Viruega et al., 2013). In addition, conidia are spread over longer distance by the psocoptera *Ectopsocus briggsi*, which carries them on body surface or in excrements (De Marzo et al., 1993).

Germination of *V. oleaginea* conidia is optimal at 16-21°C but can occurs in a wide range of temperature (3-25°C) provided there is free water; conidia start to germinate after 6 hours in optimal conditions (Obanor et al., 2008b; Agosteo and Schena, 2011). The optimal temperature for infections is around 20°C in the case of short period of wetness (less than 24 hours) while lower temperature (around 15°C) is preferred with longer wetness durations (Viruega et al., 2011; Gonzalez-Dominguez et al., 2017).

*V. oleaginea* forms appressoria and infects olive tissues by direct penetration of the cuticle with the hyphae (Obanor et al., 2008b; Lanza et al., 2017). An alternative route for penetration is the foot of the trichomes present on leaf upper surface (Lanza et al., 2017). Mycelium colonizes the outer cuticular layer (**Figure 2D**) forming flat, mono-layered colonies (Graniti, 1962; Graniti, 1965; Agosteo and Schena, 2011; Lanza et al., 2017). During the colonization, the pathogen degrades and uses cutin, wax, lipoids, cellulose and pectin as food sources (Agosteo and Schena, 2011).

After infection, the duration of the latency period is greatly influenced by temperature and humidity conditions. In the Mediterranean climate, cold winter temperatures and dry/hot conditions typical of the summer period prevent the development of the pathogen within leaf tissues and causes long periods of latency (Viruega et al., 2013). For instance, Roubal et al. (2013) reported latency periods of 16, 60, and more than 120 days at 16, 6, and 25°C, respectively. Consequently, new infections and sporulation occur during spring and autumn while latent infections enable the overwintering and oversummering of the pathogen (Graniti, 1993; Viruega et al., 2013). In the northern olive growing areas like central-northern Italy, there is a long latency during winters due to low temperatures while, in areas characterized by mild winter like southern Italy, southern Spain and north Africa the winter latency may be very short or inexistent and prevails the summer latency.

## 4 Diagnosis

When classical symptoms and signs of OLS disease are present on olive trees, diagnosis is rather easy and can be confirmed by microscopic observations of conidia and conidiophores. By contrast, the difficulties arise with the detection of asymptomatic latent infections (Graniti, 1993; Viruega et al., 2013). Loprieno and Tenerini (1959) developed a simple method based on the observation of round black spots after the leaves are immersed in a sodium or potassium hydroxide solution. However, this method is not very sensitive as it detects the disease only if the pathogen is at an advanced colonization stage. Furthermore, it can be only used for the analysis of adult leaves while tender leaves and other olive organs including shoots, leaf stalks, fruit pedicels and peduncles, drupes and inflorescences cannot be analysed.

Recently, a molecular method based on qPCR has been developed and utilized to detect *V. oleaginea* in asymptomatic olive leaves (Scibetta et al., 2020). Authors demonstrated that very young leaves are as susceptible as the adult ones and highlighted the need for earlier fungicide treatments since leaf infections may start earlier than expected. This molecular method may be easily implemented to quantify the pathogen DNA in all olive organs, representing a valuable tool to investigate important epidemiological aspects that are still controversial within the scientific community.

## 5 Control

**Figure 4:**
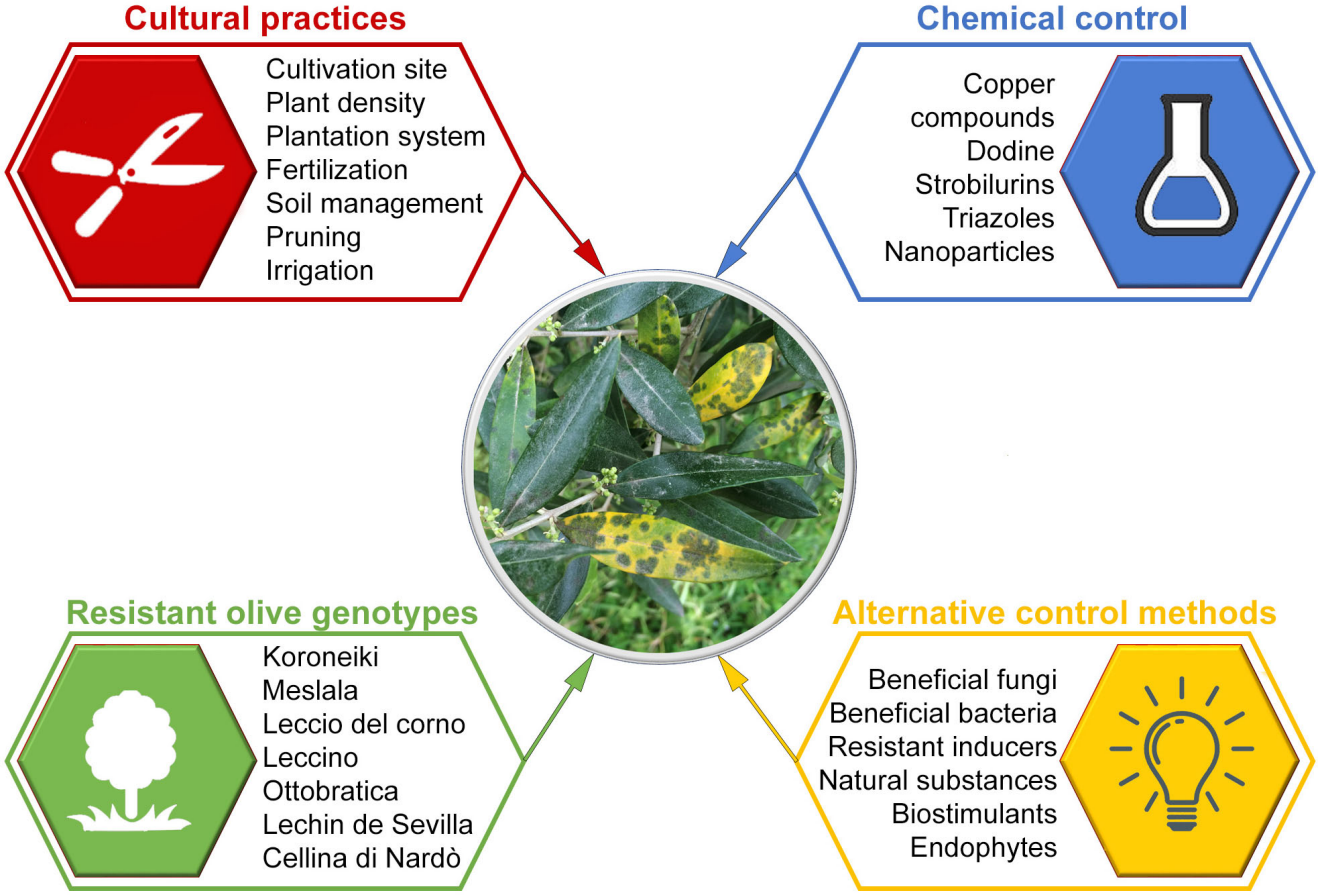
Riassuntive scheme on control of olive leaf spot caused by *Venturia oleaginea*.

Management of olive spot disease is based on the use of integrated control measures applied before and after planting (Castillo et al., 2010). They include cultural practices, resistant cultivars, chemical control, antagonistic microorganisms, natural antifungal products, and plant resistant inducers (**Figure 4**).

### 5.1 Cultural practices

Decisions made prior to planting the olive trees are essential to control the disease. The choice of cultivation site, plant density, and plantation system are very important since all conditions that reduce lightening and aeration of canopies and increase humidity and wetting period, greatly favour *V. oleaginea* infections. On hill areas the choice of sites with good exposures (south, south-west, south-east, west and east) is desirable. On the contrary, poorly ventilated or low-lying areas, low permeable soils, and land close to rivers, streams, or lakes should be avoided (Agosteo and Schena, 2011). In any case, correct soil drainage and other practices aimed at reducing soil moisture content can contribute to reducing disease incidence.

Traditional olive orchards in the majority of the Mediterranean Countries are planted at a density of about 100-300 trees per ha. During the last decades, intensive-planting- (300-400 trees/ha) and high-planting- (more than 1500 trees/ha) density orchards have been developed posing new challenges to the effective control of diseases and pests (Hall, 2011; López-Escudero and Mercado-Blanco, 2011; Connor et al., 2014). In these orchards a combination of planting design, pruning and controlled irrigation and fertilization is essential to reduce humidity and increase the efficiency of treatments by allowing the penetration of fungicides inside the canopy (Connor et al., 2014).

After planting, the cultural practices such as fertilization, soil management, pruning and irrigation have a significant influence on OLS severity. Excessive nitrogen fertilizations should be avoided since they have been associated to an increased plant susceptibility to OLS (Ouerghi et al., 2016; Roca et al., 2018) although, in a recent study, the incidence of the disease was not related to nitrogen fertilization (Rodrigues et al., 2019). In any case, a balanced fertilization is important to restrains excessive vegetative growth which can increase shading and relative humidity that favour infections. Potassium deficiency can favour OLS disease (Trapero and Blanco, 2008) and its supply was reported decrease the incidence of the disease (Ouerghi et al., 2016).

As far as soil management is concerned, the excessive growth of weeds which can sometimes reach the lower portions of canopies should be avoided. Soil tillage was associated to a reduced incidence of OLS since it prevented the excessive growth of weeds (Rhimini et al., 2020) but likely, similar results could also be obtained by mowing weeds.

Regular (annual) pruning which improves lighting and aeration is an important cultural practice for increasing lighting and reducing relative humidity and wetting periods inside the canopies (Issa et al., 2019). Pruning is also important to improve the penetration of fungicides inside the canopies during treatments.

Irrigation potentially affects the development of OLS disease by influencing the humidity of the orchard. However, no data are currently available on this practise. Nevertheless, it can be argued that management of irrigation by avoiding excessive use of water and the use of localized application (*i*.*e*., drip irrigation), along with the use of deficit irrigation, may help in reducing the incidence of OLS disease.

### 5.2 Use of resistant olive genotypes

The use resistant germplasm is considered one of the most important strategies to control OLS. For each area in which olive is cultivated, the information available regarding the most resistant cultivars and genotypes should be considered to make the best choice of the cultivar, especially when eco-sustainable cultivation methods are applied, such as the organic one. However, the choice of the cultivar is not simple since the many studies conducted in open-field conditions to identify resistant cultivars have frequently produced contradictory results. Frequently, the same cultivar is classified as resistant in a study and susceptible in another (Sanei and Razavi, 2011; Ouerghi et al., 2016; Bernaschina et al., 2020). These contradictions are mainly caused by the incorrect identification of the plant material and by differences in behaviour of clones of the same cultivar. However, other factors such as variability in virulence of *V. oleaginea* strains, and different climatic conditions in the geographical areas under study may play an important role (Agosteo and Zappia, 2007; Trapero and Blanco, 2008; Bernaschina et al., 2020). In the last decade, molecular investigations, mainly based on the analysis of microsatellites, have contributed to better define olive cultivars and should greatly facilitate the future selection of cultivar resistances to OLS (Muzzalupo et al., 2014; Gómez-Rodríguez et al., 2021). For instance the OLEA databases is a portal established in 2007 by olive researchers in Europe and contains comprehensive information about molecular and biological traits of olive cultivars including valuable data about the resistance to biotic and abiotic stress factors (http://www.oleadb.it). In **Table 1** are reported main olive cultivars with low foliar susceptibility to *V. oleaginea*.

**Table 1:** Main olive varieties with low foliar susceptibility to *Venturia oleaginea*.

Variety	% of studies * (n. of studies)
Gerboui du Nord	100 (2)
Koroneiki	100 (5)
Meslala	100 (1)
Leccio del corno	100 (18)
Leccino	90 (84)
Ottobratica	90 (20)
Lechin de Sevilla	88 (18)
Cellina di Nardò	88 (18)
Ravece	87 (8)
Pisciottana	80 (15)
Grossa di Cassano	72 (11)
Itrana	66 (9)
Dolce Agogia	65 (20)
Maurino	58 (31)
Ogliarola di Lecce	57 (14)
Sant'Agostino	55 (20)
Haouzia	50 (2)
Caiazzana	50 (4)
Arbosana	50 (2)
Ascolana tenera	48 (43)
Nostrale di Rigali	38 (13)
Ascolana dura	37 (8)
Coratina	36 (25)
Canino	35 (14)
Kalamata	33 (3)
Blanqueta	33 (3)
Lechin de Granada	28 (7)
Rotondella	25 (4)
Pendolino	20 (25)
Ogliarola barese	15 (20)
Bella di Spagna	12 (8)
Frantoio	12 (73)
Carboncella	12 (31)
Arbequina	5 (18)

*% and number of scientific studies in which the variety was reported to have low susceptibility to the disease. Data obtained by OLEA database: http://www.oleadb.it.

Studies to select and produce new varieties resistant to OLS through breeding programs have been very limited. As a result, only very few resistant genotypes have been produced (Lavee et al., 1999; Lavee et al., 2004). Further genomic (Guerin et al., 2002; Mekuria et al., 2002) and transcriptomic (Benitez et al., 2005) studies will be fundamental to identify genetic traits useful to assist breeding programs for OLS resistance.

In a breeding program, it is of paramount importance to know the resistant/susceptibility mechanisms to a plant disease. Among the structural pre-existing mechanisms, a high thickness of the cuticular layer (Petri, 1913; Agosteo and Schena, 2011; Ouerghi et al., 2016) and thinner palisade cells (Morettini, 1954) were correlated with greater resistance to OLS. The comparison of different cultivars (Chetoui, Picholine, Arbequina, and Meski) suggested that a higher incidence of OLS disease tends to be associated with thinner cuticle and higher trichome density and diameter (Ouerghi et al., 2016). All that is consistent with the ontogenic resistance observed in olive leaves to OLS disease. In fact, young olive leaves are less cutinized respect to older ones and are more susceptible to OLS (Graniti, 1993; López-Villalta, 1999; Sergeeva et al., 2009; Viruega et al., 2011; Sanei and Razavi, 2011; Ouerghi et al., 2016). This observation is further strengthened by recent results obtained by Scibetta et al. (2020) through a molecular approach, who demonstrated that very young olive leaves are susceptible to OLS disease, while in the past they were considered immune or at least less susceptible to infections (Peglion, 1928).

As concern the chemical mechanisms, Iannotta et al. (2007) reported a negative relationship between the susceptibility to *V. oleaginea* infections and the level of oleuropein in the leaves. El Aabidine et al. (2010) demonstrated that tyrosol and its derivatives were associated with preexisting defenses, whereas oleuropein and rutin were correlated with induced defenses. Authors speculated that the biosynthetic pathways of latter phenolic compounds and/or the expression rate of the corresponding genes could be at the origin of the degree of resistance of olive tree/varieties to OLS disease. Rahioui et al. (2013) reported faster and more intense PAL (phenylalanine ammonia-lyase) activity and accumulation of soluble and parietal phenols in leaves of a resistant cultivar as compared to a susceptible one.

### 5.3 Chemical control

Chemical control of OLS disease is mainly carried out with contact copper compounds and to a lesser extend with cytotropic-translaminar (dodine and strobilurins) and systemic (triazoles) fungicides (Obanor et al., 2005; Roca et al., 2007). Copper compounds, such as bordeaux mixture, copper oxychloride, copper hydroxide, copper oxide and tribasic copper sulphate, have a double action: prevention of the pathogen attacks on healthy leaves and reduction of the inoculum for new infections (Iannotta et al., 2002; Obanor et al., 2008a; Belfiore et al., 2014). In fact, copper can penetrate leaves through wounds caused by erupting conidia during the evasion and being copper phytotoxic, causes the premature drop of leaves (Graniti, 1993). As a result, there is a reduction of the inoculum available for new infections as the masses of conidia on the fallen leaves are no longer available for further infections (Agosteo and Schena, 2011; Trapero and Blanco, 2008). Since the efficacy of copper is strictly related to the disease cycle of the pathogen (eruption of conidia), the use of predictive models to determine frequency and timing of fungicide applications would be desirable (Thomidis et al., 2021). However, the use of predictive model is still very limited and treatments are empirically made in autumn and/or spring in most olive-growing countries (Agosteo and Schena, 2011).

In recent years the use of copper compounds in agriculture have raised concerns for the high level of accumulation in the soil, the risk of surface water contamination, and the potential public health problems due to the entering of copper in the food chain (Lamichhane et al., 2018). As a consequence, the European Union has restricted the use of copper compounds prescribing a maximum of 28 kg/ha of metallic copper over a period of 7 years (on average 4 kg/ha/year) and further restrictions are expected in the near future. To optimize OLS control with copper fungicides, it is therefore advisable to use: i) formulations with high efficacy and lower copper amount; and ii) rate and spray volumes calculated on the basis of tree canopy- or tree-row-volumes (Lamichhane et al., 2018).

In comparative OLS disease control trials in which copper hydroxide, copper sulfate, copper oxide, or copper oxychloride were applied in autumn and spring (3-4 applications), the best efficacy was obtained with copper hydroxide, which provided a relatively low content of metallic copper (Pasqualini et al., 2020). Interesting results have been recently obtained with a new formulation containing copper complexed with gluconate and lignosulphonate (Disper Cu Max^®^-Eden Modern Agriculture S.L-Spain), which, in spite of the lower content of metallic copper (e.g. five times lower than copper hydroxide), proved more effective than traditional formulations (Almadi et al., unpublished; Adawi et al., 2022). The higher efficacy was associated to a supposed cytotropic phytomobility of the formulation, which may also exert a curative action. Recently, nanoparticles appeared particularly promising to improve the efficacy and reduce the impact of control strategies against plant diseases (Ungureanu et al., 2021; Balestra and Fortunati, 2022). In particular, copper nanoparticles were more active in reducing *V. oleaginea* mycelial growth and OLS incidence compared with copper oxide and copper hydroxide (Ntasiou et al., 2021).

Among the curative fungicides, dodine has been reported to be as effective as copper compounds (Iannotta et al., 2002; Belfiore et al., 2014; Lahoz et al., 2014), or better when used in highly infected trees, as a result of its curative effect. In a recent study conducted on olive trees with a high latent infection incidence, dodine was more effective than copper in reducing new infections, the appearance of symptomatic leaves and the defoliation (Almadi et al., unpublished). In particular, the use of dodine against the autumnal attack of *V. oleaginea*, which causes heavy defoliation in the successive spring, allowed most of the old leaves to remain on the tree until the new ones had formed (Almadi et al., unpublished). Authors highlighted that this was important to supports the olive growth processes in the early part of the growing season and increased the growth of inflorescences (Almadi et al., unpublished). In Italy, protocols for integrated pest management specify only one treatment/year with dodine, however its application both in autumn and at the end of winter – beginning of spring may be advisable in the case of heavy autumnal infections (Almadi et al., unpublished). In this way dodine is used once per year, but with a strong synergic effect. Heavy treatment schedules should be aimed to reduce year-to-year the inoculum of *V. oleaginea* in order to progressively reduce the number of future treatments.

As concerns others curative fungicides, interesting results were obtained with strobilurins and triazoles. In particular, high protections were obtained with tebuconazole + trifloxystrobin (D'Ascenzo et al., 2014; Vizzarri et al., 2014; Najafi et al., 2021), azoxystrobin (Zuffa and Ricci, 2018), difenoconazole + azoxystrobin (Almadi et al., unpublished), and kresoxim-methyl (Viruega et al., 2002; Trapero et al., 2014), kresoxim-methyl + copper sulphate (Obanor et al., 2008a).

### 5.4 Alternative control methods

Replacing fungicides with other less harmful products for controlling OLS disease is a major challenge especially in organic agriculture. Some bacteria (*Pseudomonas*, *Bacillus* and *Microbacterium*) and fungi (*Alternaria*, *Aureobasidium* and *Phoma*) isolated from olive leaves were able to reduce the germination of conidia of *V. oleaginea* (Roca et al., 2009; Al-Khatib et al., 2011; Salman, 2017). Other antagonists such as *Pseudomonas fluorescens* (ORS3), *Bacillus atrophaeus* (BAT), and *Bacillus amyloliquefaciens* (FZB24) proved also effective in natural field conditions (Salman, 2020; Tziros et al., 2021). In recent years the investigation of epiphytic and endophytic microbial populations (microbiome) associated to olive trees has increasingly appeared as strategic to develop effective biological control strategies (Abdelfattah,et al, 2018; Abdelfattah et al., 2015; Malacrinò et al., 2022a). Indeed, the microbiome science is revealing the importance of complex network of interactions between microorganisms within each ecological niche, suggesting that a single microbial isolate might not be sufficient to contrast the development of plant pathogens. In this context, synthetic microbial communities (SynComs) defined as small consortia of microorganisms designed to mimic, at some scale, the observed function and structure of the microbiome in natural conditions might be the key to develop the next generation of effective and sustainable management strategies (Malacrinò et al., 2022b).

A number of natural substances have been assayed to control OLS disease. Rongai et al. (2012) reported the use of a *Brassicaceae* meal in vegetable oil to protect olive trees. Five applications of this formulation (2 in spring and 3 in autumn) provided a protection comparable with that exerted by dodine. Treatments with a pomegranate peel extract (PGE) few days before full boom significantly prevented latent infections over the summer period and reduced the premature defoliation of plants (Pangallo et al., 2022). Promising results were also obtained with aqueous leaf extracts of *Ambrosia artimisiifolia* (Kleef and Salman, 2021).

Another possibility for controlling plant diseases is the exploitation of plant induced systemic resistance (Buonaurio et al., 2009; Walters and Fountaine, 2009). In pot-cultivated olive plants, promising results were obtained with a number of plant resistance inducers, applied as foliar spray or soil drench: acibenzolar-S-methyl, 3-amino butyric acid, chitosan, harpin, laminarin, phosphates (FoliR-Fos 400^®^), salicylic acid and silicon (Actisil^®^) (Obanor et al., 2013; Nascimento-Silva et al., 2019; Tziros et al., 2021). Recently, in an open-field trial, a very good protection against *V. oleaginea* attacks, comparable to the dodine, was demonstrated using the self-defense inducer Disper Broton GS^®^ (Adawi et al., 2022). This formulation also contains complexed copper with an amount of metallic copper 10 times lower than copper hydroxide. It is important to underline that copper compounds and others fungicides in addition to the direct antifungal activity may also act by inducing resistance in the host (Hammerschmidt and Kuc, 1995; Gozzo and Faoro, 2013). Similarly, biological control agents such *Bacillus amyloliquefaciens* and plant extracts such as PGE are known to induce resistance in plants (Chowdhury et al., 2015; Belgacem et al., 2021).

Finally, worth of mentioning is also the use of biostimulants, which include microbials, botanicals and chemicals increasingly used in agriculture for their beneficial effects on plant growth and health (Corsi et al., 2022). Recently, they have been also used in oliviculture to promote the growth and to protect olive trees from Verticillium wilt (Almadi et al., 2020; Regni et al., 2021; Lopez-Moral et al., 2021).

### 5.5 Decision tools for OLS disease control

Chemical control of OLS disease is traditionally performed according to a calendar of applications and spring and early autumn are indicated as critical periods for disease control in many Countries where olive is grown (Obanor et al., 2008a; Viruega et al., 2011). However, the rational control of plants pests should be based on integrated pest management (IPM), which is fundamental for ensuring agricultural productivity while maintaining economic and environmental sustainability (Rossi et al., 2019). IPM is based on eight principles: 1) prevention of pest occurrence and suppression of pest populations; 2) monitoring of harmful pests; 3) informed decision-making; 4) priority to non-chemical methods; 5) multi-criteria selection of pesticides; 6) pesticide use reduction; 7) avoidance of pests resistance to pesticides; and 8) evaluation (Barzman et al., 2015). Plant protection models and other decision tools have assumed a key role in supporting decision-making process.

Viruega et al. (2011; 2013) and Obanor et al. (2011) studies have significantly contributed to develop OLS disease epidemiological models, determining the influence of a number of host, pathogen and environmental factors on *V. oleaginea* infections. Roubal et al. (2013) developed empirical models essentially based on rain events, air temperature and duration of relative humidity. Thomidis et al. (2021) studied the effect of temperature and leaf wetness on conidial germination of Greek *V. oleaginea* isolates and verified the validity of the generic and polynomial models developed by Magarey et al. (2005) and Obanor et al. (2011), respectively. The generic model predicted lower severity, which fits well with the incidence of the disease symptoms on unsprayed trees. In contrast, the polynomial model predicted high severity levels of infection, but these did not fit well with the incidence of disease symptoms.

Respect to empiric models, mechanistic ones are highly reliable in investigating how biotic and abiotic drivers influence individual’s life-history and, in turn, the overall population dynamics/epidemics (Rossi et al., 2019). A weather-driven, eminently mechanistic and dynamic model, denominated SCABOE (scab of *Olea europaea*) has been constructed on the basis of the literature data to simulate the ecological behavior of OLS disease (Rodríguez, 2017; Romero et al., 2017).

SCABOE represents a good model for scheduling treatments with chemical or biological formulations in olive orchards, but it still requires further validation in different olive producing location and countries. In particular SCABOE should be improved through the acquisition of further knowledge on the effect of *V. oleaginea* on leaf fall (Romero et al., 2017), which could also contribute to the intervention threshold determination. In fact, in experiments aimed at evaluating the effect of artificial defoliation on photosynthetic activity, it has been demonstrated that a reduction in leaf surface area up to 20% can be compensated by an increase in the photosynthetic activity of the remaining leaves (Proietti et al., 2006). Hence, it can be argued that when the percentage of symptomatic + asymptomatic infected leaves exceeds this value, treatments should be performed in order to keep the disease under control.

## 6 Conclusions

The wide spread of OLS disease in all olive cultivation areas renders this disease one the first causes of yield losses in olive orchards also in those countries where olive has been recently introduced (Sergeeva et al., 2009; Agosteo and Schena, 2011; Yesica et al., 2019). OLS damages are probably underestimated as early leaf symptoms are difficult to distinguish and the phylloptosis of diseased leaves creates the illusion that the plant is healthy or restored. Therefore, this disease deserves more attention also in consideration that the increased consumption and demand for olive oil is leading to a gradual transition from traditional plantation systems to high density and super-high-density systems (Tous et al., 2010), where the risk of OLS occurrence is higher (Rallo et al., 2013; Connor et al., 2014).

OLS is mainly controlled by copper compound applications wherever the olive is cultivated. However, according to the regulation 2009/1107 of the European Commission, copper compounds are included in the list of substances candidates for substitution as they are persistent in soil and toxic for the environment. In fact, the half-life in soil is greater than 120 days and the long-term no-observed effect concentration for aquatic organisms is less than 0.01 mg/L. Furthermore, copper accumulation can contribute to human aging and increase the risk of Alzheimer’s disease (Coelho et al., 2020). For these reasons, the allowed quantity of copper has been progressively reduced to 28 kg per hectare over a period of 7 years and its authorization will expire on December 31, 2025 (European Commission implementing regulation 2018/1981). It is therefore urgent to find alternative control means, exploiting for example nanotechnology, endophytic microbes and biostimulants (White et al., 2019; Fu et al., 2020). Furthermore, it will be important to improve and validate SCABOE and other similar mechanistic epidemiological models to predict infection of *V. oleaginea*, optimise control strategies, and reduce fungicide applications. In this context, the application of sensitive molecular detection methods is strategic to acquire information and improve the predictive models (Scibetta et al., 2020). All these, are important steps towards the implementation of sustainable agriculture systems and are particularly relevant in a period where climate changes is having a heavy impact on agriculture, plant diseases, and olive cultivation (Fraga et al., 2021).

## Author contributions

All authors contributed to the article and approved the submitted version.
